# Altering of the sprayed wall after indoor residual spraying and associated factors among households in Boricha district, Sidama regional state, Ethiopia, 2019: community-based cross-sectional study

**DOI:** 10.1186/s12936-023-04573-8

**Published:** 2023-05-02

**Authors:** Medina Ibrahim, Hunachew Beyene, Alemu Tolcha, Habtamu Eskendir, Abiyu Ayalew Assefa

**Affiliations:** 1 Department of Public Health, Hawassa University Comprehensive Specialized Hospital, Hawassa University, Hawassa, Ethiopia; 2 Department of Environment Health, College of Medicine and Health Science, Hawassa University, PO. Box 1560, Hawassa, Ethiopia; 3 Department of Public Health, Hawassa College of Health Science, P.O. Box: 84, Hawassa, Ethiopia

**Keywords:** Wall alteration, Boricha district, Ethiopia, IRS, Vector control

## Abstract

**Background:**

Indoor residual spraying (IRS) has been the main tool used to control malaria. Reducing the life span and the density of the vector mosquitoes are direct effects of IRS towards restricting malaria transmission. Residents must not wash or re-plaster walls after the spray application for at least 6 months to fight against malaria with IRS. This study sought to assess the alteration of the sprayed wall after the IRS operation and associated factors among households in the Boricha district.

**Methods:**

Community-based cross-sectional study was conducted among 608 households selected using multi-stage sampling. A structured interviewer-administered questionnaire was used to collect data. Data were analysed by SPSS version 25. Both bivariable and multivariable logistic regression analysis was done. Finally, the strength of the association was measured based on AOR with 95% CI and statistical significance was declared at a p-value less than 0.05.

**Result:**

From the total of 608 sprayed houses included in the study, 37.3% (95% CI: 33.41% – 41.15%) were found to have altered sprayed walls. The highest class of wealth index category (AOR = 2.50; 95% CI: 1.19, 5.16), low level of comprehensive knowledge about IRS (AOR = 6.08; 95% CI: 3.37, 10.94), did not get information within 2 weeks before spray (AOR = 2.09; 95% CI: 1.43, 3.05), absence of supervision after the spray operation (AOR = 1.77; 95% CI: 1.27, 2.73) and walking distance to nearest health facility (AOR = 2.39; 95% CI: 1.63, 3.35) remained significant factors of altering of the sprayed wall after IRS.

**Conclusion:**

The prevalence of alteration was relatively high. The highest socio-economic status, poor knowledge about indoor residual spraying, lack of information about IRS within two weeks before spray, absence of supervision after IRS, and walking distance of more than 30 min to reach the nearest health post were the factors affecting the alteration status of the sprayed wall. Future efforts to focus on successive awareness creation activities should be done before and after IRS operation to the community by concerned bodies.

## Introduction

Malaria is the most serious mosquito-borne illness that affects people and is regarded to be life-threatening, which is contracted through the bite of an infected female Anopheles mosquito [1]. Globally, an estimated 3.3 billion people living in 109 countries are at risk of contracting malaria disease [2]. According to the latest global malaria reports, in 2020, an estimated 241 million new cases and 627,000 deaths of malaria, of which 96% of global malaria cases and 98% of malaria deaths occur in Africa. In Ethiopia, 1.8% of cases and 1.5% of deaths were documented in 2020 [3].

Approximately 60% population of Ethiopia lives in malaria-endemic areas having altitudes below 2000 m and the majority of malaria cases are caused by *Plasmodium falciparum* and *Plasmodium vivax*, accounting for 60% and 40% of all cases, respectively. Ethiopia is among the few countries with unstable malaria transmission, which peaks bi-annually from September to December and April to May [4, 5]. Sidama regional state is one of the regions of Ethiopia where a large number of malaria episodes occurred. As one of the malarious districts of the Sidama region, the 2018 malaria report revealed malaria was the second leading cause of morbidity and annual parasite incidence (API) was 11.8 in the Borecha district [6].

Despite having a goal to eliminate malaria by 2030, Africa is not on track as the continent did not achieve its target of reducing malaria incidence and mortality by 40% by 2020 [7]. Ethiopia has already started an elimination programme in two hundred thirty-nine (239) districts to achieve the goal stated by the country by 2030 [8]. Several vector control interventions have been put in place to prevent malaria in Ethiopia, the key interventions are indoor residual spray (IRS), long lasting insecticidal nets, Early Diagnosis and Case Management, Surveillance and response, Community empowerment and mobilization lastly monitoring and evaluation [9].

According to the new Federal Ministry of Health, malaria risk stratification, 14.8% of the country’s total population is targeted for IRS [10].

In Ethiopia, efforts are being made toward achieving the desired plan of the national malaria control programmes. IRS involves spraying internal walls and ceilings of dwellings using insecticides with residual action (i.e., insecticides that remain on the surface for a long time). The effectiveness of this control method depends to a large extent on the vector’s sensitivity to the insecticide used, the quality of insecticide dosage on the spray-able surface area during operational work, and how much they like to rest indoors. Most vectors in Africa prefer to rest indoors [11].

Though the malaria prevention and control programme in the country has employed several organizational approaches, from the highly centralized vertical malaria eradication setting to an integrated and decentralized approach, IRS remains a key component of the national malaria prevention and control strategy since the 1950s [12, 13]. The basic principle behind IRS is that, after biting, the female mosquito eventually rests on sprayed surfaces of the house, where they come into contact with the residual (long-lasting) insecticide sprayed on walls and furniture, and they die within a few hours, thus it reduces the life span of vector mosquitoes which in turn prevents transmission of the parasite to others [14, 15]. The walls are sprayed with 0.4 g/m^2^ and 2 g/m^2^ Bendiocarb and Propoxur respectively. The residual efficacy of propoxur was recorded at > 80% at week 17 on standard spray regardless of the wall types while it was < 80% on routine spray except on painted wall surfaces [14]. Some insecticides also irritate mosquitoes and cause them to leave houses before biting, as a result, it reduces human-vector contact [15].

The residual life of the insecticide on sprayed surfaces varies between different chemicals, if peoples use IRS correctly the life span of the insecticide at the household (HH) level is usually between 4 and 6 months. In target areas, IRS coverage of 85 percent or more leads to the maximum protection for the population and can interrupt transmission in the immediate area [16].

In 2020, 2.6% and 5.3% of the population were protected by IRS globally and in Africa, respectively [2]. A study conducted in Kenya revealed a marked decline in the monthly testing positivity rate was shown in a place where IRS was taken as an intervention strategy [17].

Despite having been conducted every year and having coverage of 90% IRS [6] among highly malarious kebeles of the district, there is still a rise of reported cases among the sprayed kebeles. Furthermore, data about the alteration of the sprayed wall is also scarce, particularly in the study area. Therefore, this study aimed to assess the magnitude and factors of altering the sprayed wall after IRS among households in the study area.

## Methods and materials

### Study area

The study was carried out in Sidama regional state, Boricha district which is found south of Hawassa city. Based on the central statistic estimate the total population of the district for the year 2019 GC is 332,791 (male 167,893 female 164,898) pregnant mothers 11,555 and under-five children 50,451). These populations are distributed in 39 rural and 5 urban kebeles. Concerning the climatic condition, the district is characterized by kola 87% and woinadaga 13%, and the district is labelled as malarious since the altitude falls below 2000 m above sea level.

Concerning the health care services, there are 1 primary hospital, 10 health centres, 39 health posts, and 8 private clinics. As there is an intense transmission of malaria in the district, curative and preventive actions are actively undergone. Among these, IRS was done in selected rural kebeles once a year, The district health office’s annual report in 2018 showed that approximately 174,450 ITNs were distributed for 67,916 households who are living in 42 kebeles and 21,006 HH was sprayed from 11 kebeles and this made IRS coverage 40% from all kebeles households and 90% from sprayed kebeles households [6].

### Study design and study period

A community-based cross-sectional study was conducted from March 8 to April 9, 2019.

### Populations

All sprayed households in the district were the source population while households found in randomly selected five sprayed kebeles were the study population. Randomly selected households from which actual information was collected were study units.

### Eligibility criteria

Houses in the selected kebele which have permanently residing household heads during and after the IRS spray were included in the study while houses whose heads of the HH were absent after repeated visits, unable to listen, talk, and critically ill during the data collection period were excluded from the study.

### Sample size determination

The sample size was determined using single population proportion formula (n = (Zα/2)2 p (1 − p)/d2)) by considering the following assumptions: proportion of households that altered sprayed wall after IRS = 50% (assumed), 95% confidence interval, 5% margin of error. After using design effect 1.5 (since a multi-stage sampling technique was used) and a possible non-response rate of 10%, thus the final sample size required for the study was found to be 633 HH.

### Sampling procedures

Multi-stage sampling technique was carried out. Primarily, 11 IRS-sprayed kebeles were selected, then 5 (45%) kebeles out of 11was selected by simple random sampling methods, Where IRS has been ongoing. Secondly, for the sampling frame, the registered households were identified using registries that have lists of sprayed households in the kebele health post. Then, by using systematic random sampling every fourteen (K = 14) households was incorporated into the sample. The numbers of households included in the study from each kebele were determined proportionally based on the population of each kebele. A representative sample of 633 households was selected.

### Variables of the study

Altering of the sprayed wall was a dependent variable while demographic characteristics (age, gender, socio-economic situations, educational level, religion, and housing structure), behavioral factors (knowledge about malaria transmission and knowledge about IRS), environmental factors (presence of pond near to house, stagnant water and housing structure), health-related factor (source of information, use of another intervention, supervision, and message during and after spraying, experience of using IRS and malaria disease history) were independent variable of the study.

### Operational definitions

Altering of the sprayed wall: in this study altering the sprayed wall is the act of washing, brushing the wall, covering it with cloths, hanging posters and photos to decorate the house, and re-plastering any part of the insecticide-sprayed wall, with mud, dung or paint by householders before the end of potency period (6 months) of the sprayed insecticide.

Indoor residual spraying: the application of long-acting chemical insecticides with a residual effect on the walls and roofs of all houses and domestic animal shelters in a given area, to kill the adult vector mosquitoes that land and rest on these surfaces.

Insecticide spraying season: a time assigned for insecticide spraying ahead of the arrival of the malaria transmission season, but calculated to maintain the efficacy of the insecticide sprayed up to the end of malaria transmission season of that particular place.

Household head: any person either male or female who owns or rents a particular house and decides for the entire family.

Comprehensive knowledge level about IRS: household heads who correctly responded to 75% or above, between 50–74%, and 49% or below on the knowledge assessment questions were categorized as having high, medium, and low comprehensive knowledge about IRS, respectively [18].

Thatched or grass-roofed: these types of houses are more convenient for mosquitoes to enter the room and hide between logs and grass layers causing an increased risk of mosquito bites to those who live in thatched roof houses.

Rough wall surfaces: wall types that are suitable for mosquitoes to hide during the day.

### Data collection instrument and procedures

Data was collected using structured interviewer-administered questionnaires. The questionnaire incorporates different contents which include socio-demographics, household head knowledge about malaria, knowledge about IRS, health service-related factors, environmental factors, alterations made on the walls after IRS, and factors associated with alteration of indoor residual spraying. The questionnaire was developed by reviewing similar literatures [12–14, 17–19] and adapted to fit the aims of this study.

The data were collected by five health workers (two of them are a nurse and three of them are environmental health). Remarks were given during the morning times on how to eliminate or minimize errors and take corrective actions timely. Based on the sampling procedure in the households either the head of the house/Father or mother from selected houses was interviewed. Furthermore, an inspection was made for a few questions which need further confirmation by trained data collectors.

### Data quality assurance

The questionnaires were first prepared in English and translated to the local language “Sidamu afoo” and later translated back to English to ensure reliable information. Two days of training were given to data collectors on the aim of the study, the content of the questionnaire, and how to collect the data. Questionnaires were translated and pre-tested on 5% of the total sample size in the nearby district. Accordingly, necessary corrections were made to the questionnaire before the actual data collection. Close supervision and spot-checking for the filled and returned questionnaire for accuracy and completeness of data was done on daily basis.

### Data processing and analysis

Data were entered using Epi-info7 and exported to the statistical package for social sciences (SPSS) version 25 for further analysis. Frequencies and cross-tabulations were used to check for missed values and variables. Descriptive statistics were computed to describe the study subjects. Crude and adjusted odds ratios from bivariable and multivariable analyses respectively were used to measure the association between variables. Bivariable analysis with a P-value < 0.25 variables were selected as candidate variables for the multivariable logistic analysis. The goodness of fit was tested by the Hosmer–Lemeshow statistic which is not significant P-value = 0.76 and Omnibus tests which is significant P-value < 0.001. Multivariate logistic-regressions were used to adjust for possible confounding variables. An adjusted odds ratio with 95% CI and P-value < 0.05 was computed to assess the strength and significant level of the association.

Regarding the Wealth index of the family, a single variable denoting the economic status of the household was created, termed the ‘wealth index’ overall to select variables that best explained the wealth variance. In the first step, 11 variables that measure wealth were selected. These included availability of agricultural land, number of cattle, number of sheep or goats, number of donkeys or horses, ownership of radio, and type of house (roof made from thatch or corrugated iron sheets). Grain, coffee, vegetables were scored. Score 0 if the variables are not available and 1 = if the variables were available. To do that, a principal component analysis (PCA) was done and all assumptions should be satisfied like sample size (valid cases) should be 50 or more here is 608, the ratio of cases to variables at least 5 to 1 here is 608/11 = 55 (55 to 1), there should be more than two correlations that have value 0.30 or greater measure of sampling adequacy should be greater than 0.50, Probability for Bartlett test of sphericity should be less than the level of significance here and Kelvin–Meyer–Olkin test (KMO) were not significant and communality for each variable should greater than 0.50 for this study variables with value for less than 0.05 were removed as lowest communality and analysis were repeated. After the assumptions required or the PCA characteristics were combined to produce a single variable of wealth quintiles and used for further analysis involving the calculated wealth of specific households.

## Results

### Socio-demographic characteristics of the respondents

From the total of 633 households visited, complete responses were obtained from 608 which makes the response rate to be 96.8%. Four hundred eighty-four (79.6%) of the household heads include in the current study were males. Regarding educational status, 187 (30.8%) of the respondents can at least read and write during the survey period. The majority (86.3%), of the respondents, were married. The predominant religion of the respondents was Protestant 504 (82.9%); followed by Muslim 93 (15.3%). Sidama is the dominant ethnic group among the respondents constituting 597 (98.2%) of the total participants there were 525 (86.3%) farmers and others (including merchants, and daily labourers) (Table 1).

**Table 1:** Socio-demographic profile of the survey participants, Boricha District Sidama regional state, Ethiopia, 2019

Variables	Frequency	Percent
Gender of HH head		
Male	484	79.60
Female	124	20.40
Age		
15–24	22	3.60
25–34	176	28.90
35–44	220	36.20
45–54	120	19.70
≥ 55	70	11.50
Marital status of HH head		
Married	561	92.30
Single	28	4.60
Others^a^	19	3.10
HH head occupation		
Farmer	525	86.30
Other^b^	83	13.70
Wealth index		
Lowest	125	20.60
Second	122	20.10
Middle	71	11.70
Fourth	173	28.50
Highest	117	19.20

^a^Other: widowed, divorced

^b^Other: merchants, daily laborers, government employee

### Household heads knowledge about malaria

All household heads had heard about malaria. When asked about the main cause of malaria, 516 (84.80%) responded it is caused by mosquito bites. Different malaria prevention measures were stated by respondents, ITNs use by 478 (78.60%), IRS by 346 (56.9%), drainage of stagnant water by 117 (19.2%), take tablets by 96 (15.6%) and making the house warm by 52 (8.6%) using boiled water by 22 (3.6%) of the respondents When asked about resting place of mosquitoes, 224 (40%) of the respondent answered mosquitoes mostly rest; on walls inside the house, 245 (40.5%) outside the house 224 (36.5%), 147 (24.2%) in the water body and 7 (1.2%) in the air (Table 2).

**Table 2:** Household heads knowledge of the cause, preventive measures and resting place of malaria in Boricha District, Sidama regional state, Ethiopia, 2019 (n = 608)

Variables	Frequency	Percent
The main cause of malaria		
Mosquito bite	516	84.80
Cold	37	6.60
Drainage of stagnant water	35	6.20
Rain	20	3.70
Malaria preventive measure (n = 608)^b^		
Use insecticide-treated nets (ITNs)	478	78.60
Use indoor residual spraying	346	56.90
Drainage of stagnant water	117	19.20
Use tablet	96	15.80
Make the house warm	52	8.60
Other^a^	28	4.60
Mosquitos mostly rest on n = 608^b^		
Outside the house	245	40.30
Inside the home	224	36.80
In water body	147	24.20

^a^Other: close the door, use boiled water

^b^Multiple responses

### Knowledge about IRS

This study showed that all of the study subjects heard about IRS. Of these, 439 (72.2%) got the information from Health extension workers. The role of 1 to 5 leaders was the second most frequently mentioned source of information about the IRS, 129 (21.2%). Mass media, newspapers, and friends were mentioned as the source of this information 96 (15.7%).

When asked about how IRS or insecticide prevents malaria, 563 (92.5%) said it is by killing mosquitoes, 24 (3.9%) by repelling mosquitoes, and 19 (3.1%) by irritating mosquitoes. There were 534 (89.7%) respondents who mentioned taking materials outside the house before spraying was the role of the family members during pre-spray preparation. Other, 499 (83.9%) said their role is preparing water for chemical dilution, 15 (2.5%) and 15 (2.5%) mentioned that their role is to facilitate house members to stay outside for two hours after spaying and take sick people outside before spraying, respectively.

Regarding the potency duration of the chemical, 246 (40.5%) household heads said that the potency period of the chemical is greater or equal to 6 months, 199 (32.7%) respondents indicated that the efficacy period of the insecticide between 3 and 5 months, 153 (25%) reported that the insecticide had efficacy duration of less than three months the remaining 10 (1.6%) did not know the life span of the chemical. Three hundred twenty-seven heads of the HHs knew about malpractices that would affect the potency period of sprayed chemicals. Among them, 101 (30.9%) mentioned painting, 139 (42.5%) mentioned painting with dung, 139 (42.5%) Hanging of cloths116 (19.1%), 96 (29.4%) Painting with mud, newspaper 37 (11.3%) and 5 (1.5%) responded decorate with posters, cultural material and photos (Table 3).

**Table 3:** Household heads knowledge about Indoor residual spraying in Boricha District, Sidama regional state, Ethiopia, 2019

Variables	Frequency	Percent
Source of IRS information n = 608^d^		
Health extension workers	439	72.20
1 to 5 leaders	129	21.20
Community event	70	11.50
Other^a^	26	4.30
IRS prevent malaria n = 608^d^		
Kills mosquitoes	563	92.50
Others	45	7.50
The exact part of sprayed surface n = 595^d^		
On the surface of the inner wall	593	99.60
On the surface of the outer walls	195	32.7
Other^b^	17	2.80
Role of the family members in IRS n = 608^d^		
Removing some of the HH items from the house before spraying	534	89.70
Prepare water for diluting the chemical for the sprayer	499	83.80
IRS-friendly and unfriendly practice	29	4.90
To stay people outside for up to 2 an hour	15	2.50
Remove ill people before spray	15	2.50
Frequency of spraying n = 608		
Yearly	461	75.80
Once	68	11.10
Every six	40	6.60
Don't know	39	6.50
Once	118	19.40
Twice	171	28.10
Thrice	160	26.30
More than three	146	24.00
I don’t know	13	2.10
Potency time of the chemical on sprayed wall		
≥ 6 month	246	40.50
3 to 5 months	199	32.70
Others	163	26.80
Types of alteration will be made on the walls after IRS n = 327^d^		
Painting of walls	101	30.90
Painting with dung	139	42.50
Hanging of cloths	116	19.10
Painting with mud	96	29.40
Newspaper	37	11.30
Others^c^	18	5.47
Level of comprehensive knowledge of IRS		
Low knowledge	216	35.53
Medium knowledge	134	22.04
High knowledge	258	42.43

^a^Others: radio, friends, school

^b^Others: the surface of the earth, the inner surface of the roof

^c^Others: open door 2 h after spraying, decorate with posters, cultural material, and photos

^d^Multiple responses

### Health service related factors

Supervision was conducted for 324 (53.3%) of the households. Insecticide treated nets (ITNs) were a preferred alternative control method for 225 (37%) of households. Information before the spraying date was given for 366 (60.20%) households. 3310 persons lived in 608 HH among them 99 (2.9%) HH members were diseased with malaria after spraying (Table 4).

**Table 4:** Health related factors on alteration in Boricha District, Sidama regional state, Ethiopia, 2019

Variables	Frequency	Percent
Supervision after spraying n = 608		
Yes	324	53.30
No	284	46.70
Frequency of supervision n = 324		
Once	125	38.60
Twice	147	45.40
Thrice	52	16.00
Information within 15 days before spraying date n = 608		
Yes	366	60.20
No	242	39.80

### Environmental related factors

When asked whether the health posts were located within walking distance from the households, 284 (43.1%) replayed that they have to travel more than 30 min to reach the nearest health post. Of 414 (68.1%) houses where a mosquito breeding site existed around the residence, 330 (79.7%) houses were found within a 5-min walking distance from the breeding site. Four hundred forty-seven (76.8%) of the houses were with thatched roofs and 141 (23.3%) were roofed with the corrugated iron sheet. The walls of 571 (97.2%) houses were rough and the remaining 17 (2.8%) has smooth walls (Table 5).

**Table 5:** Environmental related factors, in Boricha District, Sidama regional state, Ethiopia, 2019

Variables	Frequency	Percent
Walking distance from the nearest health facility		
< 30 min	324	53.30
≥ 30 min	284	46.70
Presence of mosquitoes breeding site		
Yes	414	68.10
No	194	31.90
Breeding site distance from home n = 414		
< 5 min	330	79.70
≥ 5 min	84	20.30
Type of roof		
Thatched	467	76.80
Corrugated iron sheet	141	23.20
Wall types		
Rough	591	97.20
Smooth	17	2.80

### Alterations made on the sprayed walls after IRS

**Fig. 1:**
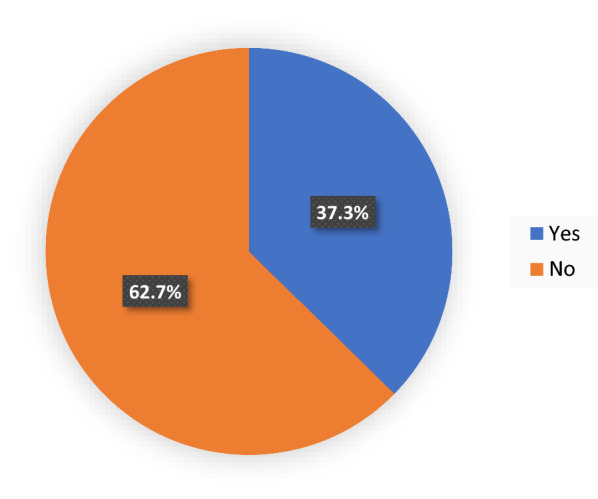
Prevalence of households by alteration made on the wall after IRS of Boricha District at household level, Southern Ethiopia, 2019

From a total of 608, sprayed houses 227 (37.3%) (95% CI 33.41–41.1%) had altered the wall during the period they should wait without touching it (Fig. 1).

### Time and types of alteration made on sprayed wall

**Fig. 2:**
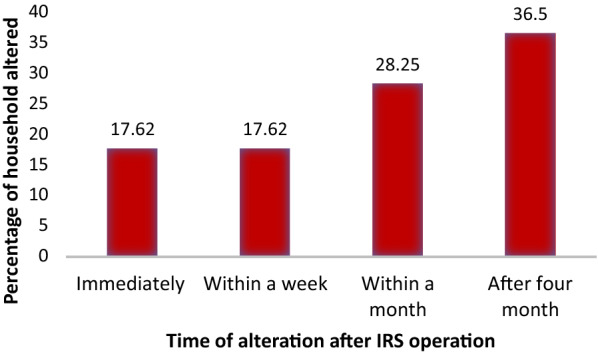
The time of alteration after the operation of indoor residual spraying at household level of Boricha District, Southern Ethiopia, 2019

**Fig. 3:**
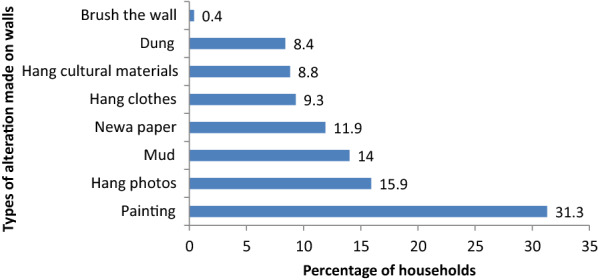
Distribution of households by types of alteration made on sprayed wall, Boricha district, Southern Ethiopia, 2019

Eighty-three houses (36.50%) altered sprayed house walls after 4 months and 40 (17.62%) immediately after the operation (Fig. 2). Types of Alteration made on walls after spraying 71 (31.3%) by painting 36 (15.9%) by hanging photos, 32 (14%) by painting with mud, plastering with news paper by 27 (11.9%) (Fig. 3).

### Reason for alteration of sprayed wall

**Fig. 4:**
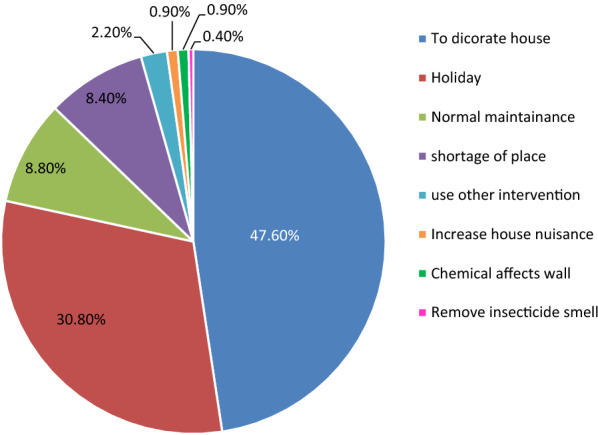
Reason for alteration reported by household heads after the operation of indoor residual spraying of Boricha District, Southern Ethiopia, 2019

The most common cited reasons why household heads alter the sprayed wall were due to aesthetic/decoration 108 (47.6%) followed by preparation for holiday 70 (30.8%). Other reasons mentioned by household heads were due to normal maintenance 20 (8.8%), shortage of place for cloths 19 (8.45%), etc. (Fig. 4).

### Factors associated with alteration of the sprayed wall after indoor residual spraying

Based on logistic regression result socio-demographic, knowledge, and health service-related factors were assumed to be associated with altering the sprayed wall (Table 6). Initially, in the bivariable analysis, eight variables with p-value < 0.25 were selected as a candidate for multivariable logistic regression. In the final model, wealth index, level of comprehensive knowledge of IRS, presence of information before spraying, and presence of supervision after spraying were found to have a statistically significant association with alteration of IRS with p-value < 0.05.

**Table 6:** Summary of logistic regression analysis on factors related to alteration of the sprayed house wall in Boricha District, Sidama regional state, Ethiopia, 2019

Variables	Wall altered		COR (95% CI)	AOR (95% CI)
	Yes	No		
Age (year)				
15–24	6	16	0.45 (0.16, 1.27)	0.69 (0.196, 2.44)
25–34	76	149	0.90 (0.52, 1.57)	0.98 (0. 47, 2.06)
35–44	71	149	0.57 (0.33, 0.98)*	0.77 (0.38, 1.56)
45–54	42	78	0.64 (0.35, 1.17)	0.77 (0.37, 1.59)
≥ 55	32	38	1	
Educational status of the respondent				
Can't read and write	181	240	2.31 (1.57, 3.40)*	1.13 (0.67, 1.88)
Can read and formal education	46	141	1	1
Wealth index				
Lowest	60	65	1	1
Second	50	72	0.75 (0.46, 1.24)	0.84 (0.45, 1.57)
Middle	22	49	0.49 (0.26, 0.98)*	1.52 (0.63, 3.66)
Fourth	49	124	0.43 (0.264, 0.69)*	1.20 (0.61, 2.36)
Highest	46	71	0.70 (0.421, 1.17)*	2.50 (1.19, 5.16)**
Comprehensive knowledge level of IRS				
Low	120	96	3.50 (2.38, 5.14)*	6.08 (3.37, 10.94)**
Medium	39	95	1.12 (0.72, 1.83)	1.6 (0.81, 2.98)
High	68	190	1	1
Information within 15 days before the spray				
Yes	111	225	1	1
No	116	226	2.2 (1.51, 2.90)*	2.09 (1.43, 3.05)**
Presence of supervision after spraying				
Yes	93	231	1	1
No	134	150	1.66 (1.19, 2.32)*	1.77 (1.27, 2.73)**
Time to reach the nearest health facility				
< 30 min	114	232	1	1
≥ 30 min	113	149	1.54 (1.12, 2.15)*	2.39 (1.63, 3.35)**
Mosquitoes breeding site				
No	139	275	1	1
Yes	88	106	0.60 (1.16, 2.32)*	1.12 (0.68, 1.85)

*COR* crude odds ratio, *AOR* adjusted odds ratio, *CI* confidence interval

NB. 1: reference, *remained significant at P-value < 0.25, **remained significant at P-value < 0.05

Those in the highest class of wealth index category were 2.5 times (AOR = 2.50; 95% CI: 1.19, 5.16) more likely to alter sprayed walls when compared with low-class families. Respondents with a poor level of knowledge about IRS in this study were six times (AOR = 6.08; 95% CI: 3.37, 10.94) more likely to alter sprayed walls compared to those with the highest level of knowledge. Those who did not get information before spraying were two times (AOR = 2.09; 95% CI: 1.43, 3.05) more likely to alter sprayed walls compared with those who did get information before spraying. Households that have not been supervised after the spray operation were 1.8 (AOR = 1.77; 95% C:I 1.27, 2.73) times more likely to alter sprayed walls compared with those being supervised. Those households who travel for more than 30 min to reach the nearest health post were 2.4 times (AOR = 2.39; 95% CI: 1.63, 3.35) more likely to alter sprayed walls compared with those who travelled less than 30 min.

## Discussion

This community-based study aimed to assess the magnitude of altering the sprayed wall after IRS and associated factors among households in the Boricha district, Southern Ethiopia. In this regard, the result of this study revealed that the magnitude of altering of the sprayed wall before the end of the potency period of the sprayed insecticide was 37.3% (95% CI 33.0%, 41.0%). This finding was in line with reports of studies conducted in South Africa, Tonga health district (34.7%) [19], and Mozambique (34.9%) [20]. However, the magnitude revealed in this study was higher than reports of previous studies conducted in South Africa (6.7%) [21], Zambia (2.1%) [22], Swaziland (10%) [23], Ethiopia Oromia Lume district (21%) [24], Eastern Ethiopia Kersa District (7.4%) [25]. The possible cause for this discrepancy might be due to the difference in socio-cultural characteristics, the health-seeking behaviour of the community, and the level of attention given to malaria intervention programmes. For example, awareness creation services before conducting of IRS and continuous supportive supervision were given by health extension workers after the operation of the IRS in Kersa district [25]. In contrast, the magnitude revealed in this study was lower than those reported in India [26] which showed a magnitude of 80%. The possible explanation for this difference could be explained by due to variability of socio-demographic characteristics and time difference while the study is conducted.

Further aim of this study was founding an association between independent variables with the dependent variables. As a result, this study indicated that wealth index, comprehensive level of knowledge, presence of information before spraying, presence of supervision after spraying, and time to reach the nearest health facility remained significant factors of altering wall after IRS. Households with a wealth index of the highest socio-economic class were above two times more likely to alter their sprayed wall after IRS compared with households with the first (poorest) socio-economic class. This evidence is supported by a previous study conducted in Equatorial Guinea [27] which indicated that wealthier respondents were more likely to be reluctant to accept IRS compared with their counterparts. Similarly, this finding lends support from a study previously done in Uganda [28], which reported that wealthier respondents were less likely to take up IRS compared with poor respondents. Furthermore, a study done in Zambia [29] showed that positive association between unemployment and the high acceptability level of the IRS. The possible explanation might be that households with the highest socio-economic level may involve in maintenance, painting, and decorating their houses frequently without considering the economic benefit of IRS, unlike households with the lowest socio-economic.

In this study, comprehensive knowledge about indoor residual spraying became an independent factor in altering sprayed walls after IRS. Those household heads who had low comprehensive knowledge about IRS were six times more likely to alter sprayed wall after IRS compared with those household heads having high comprehensive knowledge about IRS. This finding was in line with reports of a previous study conducted in Uganda [30] and the Sidama zone [18] where good comprehensive knowledge about IRS helps to demonstrate good practices of malaria prevention and control measures. Similarly, studies conducted in Nigeria [31] and Uganda [32] also supported this evidence by reporting having good knowledge about malaria prevention increases good practice of malaria prevention methods. The possible justification might be understanding the anticipated advantages these interventions would bring to their families and community in general. Furthermore, in an ideal world, good knowledge should correspond with good practice and this is what was revealed in this study. Therefore, these remind us the integration of awareness creation activities with malaria intervention programmes through community and religious leaders is vital for IRS programme efficiency.

In the current study households, who did not get information within 2 weeks before spray was two times more likely to alter sprayed walls after IRS compared with those households who got information within 2 weeks before spray. This evidence was comparable with a previous study conducted in the Lemu district of the Oromia region [24], which showed that individuals who missed the message before the IRS operation were more involved in the re-plastering of their sprayed houses than their counterparts. This finding is also supported by the evidence sought in the study conducted in Ghana [33], which indicated that having inadequate information before the IRS was a barrier to its acceptance by the community. This finding suggests combating malaria through IRS in these communities remains challenging. Therefore, advocacy and sensitization campaigns before conducting of IRS should be geared towards preparing communities for IRS to increase desired behaviour is essential.

Supervision after IRS was the other factor that affects the alteration of the sprayed wall in the current study. Didn’t get supervision after IRS were nearly two times more likely to alter the sprayed wall than those who were supervised after IRS. This result was in line with research conducted in the Oromia Lemu district where those supervised after spray were 68% less likely to re-plaster their house than those did not supervised after IRS [24]. This might be because regularly conducting supportive supervision post-IRS operation might increase their awareness which in turn decreases the possible alteration of the sprayed wall.

In this study, those households who travelled for more than 30 min to reach the nearest health facility were over two times more likely to alter sprayed walls compared with those who traveled less than 30 min from the nearest health facility. Even though there was no study to compare this specific finding, this might be justifiable in the way that the households far from health facilities were less likely visited by health extension and health workers and did not get adequate information about health-related issue compared to households near to health facility.

The findings of this study are, however, subject to some limitations. First, this study might be subjected to recall biases to recall back the date of supervision, the information they got before spraying, and the exact day of alteration. Second, there was a limited study in the area of factors related to alteration; hence some of the identified factors were not adequately compared with findings of other studies.

## Conclusion

The current study showed that the proportion of alteration of IRS on sprayed wall before the end of potency period in the area was relatively high. The two main reason for alteration mentioned by household head were aesthetic/decoration, followed by alteration for varied cermonial purposes, such as holiday and wedding.

The findings of this study also highlight that highest socio-economic status, poor knowledge about indoor residual spraying, lack of information about IRS within 2 weeks before spray, absence of supervision after IRS and walking distance more than thirty minutes to reach to the nearest health post were the factors that determine alteration status of sprayed wall. The results suggests that a need to improve households’ knowledge so as to bring behavioural change on malaria prevention methods especially on IRS along with successive awareness creation activities should be done before and after IRS operation to the community by concerned bodies. Furthermore, regular supportive supervision by district health office and health extension workers should be conducted to minimize alteration rate after indoor residual spray operation.

Finally, study should be conducted in qualitative approach to investigate in-depth information from the community on IRS and further study should be done to detect the degree of reduction in efficacy of IRS after alteration of the sprayed wall of the household.

## Data Availability

The datasets generated and/or analyzed during this study are not available for online access, however, readers who wish to gain access to the data can write to the corresponding author: Abiyu Ayalew Assefa at Email: abiyman143@gmail.com.

## Abbreviations

API: Annual parasite incidence
FMoH: Federal Ministry of Health
HHs: Households
IRS: Indoor residual spraying
ITNs: Insecticide-treated bed nets
KAP: Knowledge, attitude and practice
MIS: Malaria indicator survey
RBMI: Roll Back Malaria Initiative
SNNPR: Southern Nations, Nationalities and People’s Regional State
SPSS: Statistical Package for Social Sciences
WHO: World Health Organization
